# A novel primary antibiotic cement-coated locking plate as a temporary fixation for the treatment of open tibial fracture

**DOI:** 10.1038/s41598-023-49460-z

**Published:** 2023-12-11

**Authors:** Yongqiang Kang, Yongwei Wu, Yunhong Ma, Xueyuan Jia, Mingyu Zhang, Ming Zhou, Fang Lin, Yongjun Rui

**Affiliations:** 1 Medical College, Soochow University, Suzhou, Jiangsu China; 2 Department of Traumatic Orthopedics, Wuxi Ninth People’s Hospital Affiliated to Soochow University, Liangxi Road, No.999, Binhu District, Wuxi, Jiangsu China

**Keywords:** Health care, Medical research

## Abstract

Complex lower extremity trauma reconstruction remains a challenge. This study used an internal fixation composite structure of antibiotic cement plates as a temporary fixation to treat lower extremity Grade III open fractures; thus, reducing the treatment period and complications of external fixation. We aimed to assess the safety and efficacy of this technique in the initial surgery stage. Between January 2018 and March 2021, 20 patients with Gustilo grade IIIB/C open fractures received an antibiotic cement-coated locking plate as a temporary internal fixator during initial surgery. Thorough debridement and temporary internal fixation were performed with a 3.5-mm system antibiotic cement-coated locking plate. Ten patients required free bone fragment removal, followed by bone cement packing. The final stage involved internal fixation and wound repair with a free anterolateral thigh flap. Clinical and imaging results were retrospectively analysed. The repair time ranged 1–7 days. All flaps survived. Two patients experienced wound infection, and one developed severe bone infection 3 months after three-stage bone graft surgery. Autologous cancellous bone grafting was performed on 10 patients with bone defects 6 weeks after surgery. Bone union was universally achieved after 1 year. This method proved safe and effective, successfully repairing Grade III open fractures of the lower extremity 1–7 days post-treatment.

## Introduction

Open proximal tibial fractures commonly pose a treatment dilemma for orthopaedic surgeons, as the guiding principle in the management of open fractures is the prevention of infection^1^. However, in addition to thorough debridement, appropriate fracture fixation decreases the complications associated with open fracture management. As such, temporary external fixation is often performed when definitive primary care is not possible. External fixation may provide good temporary control, but it is generally a poor choice for definitive care. The presence of a frame may make plastic surgical reconstruction difficult, further lead to false confidence, and delay definitive soft tissue reconstruction. Current data suggests that the outcome of major limb reconstruction with an external fixator after a severe open fracture produces poor results; indeed, the LEAP study showed that the association of external fixation for bony stabilisation with soft tissue reconstruction with a flap resulted in a worse functional outcome than amputation^2,3^.

In the past, internal fixation was often avoided due to concerns about infection risk and biofilm formation^4^. However, with improved debridement techniques that ensure a clean environment, internal fixation is becoming more popular and widely accepted. Properly applied internal fixation can promote better healing and functional recovery in such situations. Excluding cases of heavy organic contamination, definitive fixation of fractures can be conducted in the upper, but not the lower, extremities^5,6^. Meanwhile, antibiotic cement-coated implants have been used in the treatment of osteomyelitis and have shown promising results^7,8^. However, its use has not been reported in the emergency department for Grade IIIB/C open tibial fractures. Thus, based on these findings, we modified the first-stage fixation by using an antibiotic cement-coated locking plate to provide stability.

In the present study, we report on a series of patients with Grade IIIB/C open fractures of the lower extremities, treated with antibiotic cement-coated locking plates as temporary fixation in the primary stage. We further investigated the outcomes of this new method to provide a reference for clinicians when applying this method.

## Methods

Between January 2018 and March 2021, 20 patients with Gustilo grade IIIB/C open tibial fractures were treated with an antibiotic cement-coated locking plate as a temporary internal fixator during the initial stage of surgery at our clinical centre. Of these, 16 and 4 were male and female patients, respectively with ages ranging from 19 to 63 years (average age, 46.6 years). In terms of aetiology, 9 injuries were caused by a machine crush, 10 were due to traffic accidents, and 1 was due to a fall. Eighteen cases were Gustilo grade IIIB, and two were Gustilo grade IIIC (Table 1). All procedures were performed in accordance with the ethical standards of the Ethics Committee of Wuxi Ninth People's Hospital (approval number KS2023009), as well as with the Helsinki Declaration of 1975, as revised in 2008. Informed consent was obtained from all patients for inclusion in the study.

**Table 1:** Statistical data of the study participants.

	Results	Percentage (%)
Patient number, n	20	
Male, n	16	80
Female, n	4	20
Mean age(range), y	46.6 (19–63)	
Gustilo–Anderson type		
IIIB, n	18	90
IIIC, n	2	10
Injury		
Machine crush, n	9	45
Traffic accidents, n	10	50
Other, n	1	5

### Ethics approval and consent to participate

All procedures were performed in accordance with the ethical standards of the Ethics Committee of Wuxi Ninth People’s Hospital (approval number KS2023009), as well as with the Helsinki Declaration of 1975, as revised in 2008. Informed consent was obtained from all patients for inclusion in the study.

## Surgical technique

**Figure 1:**
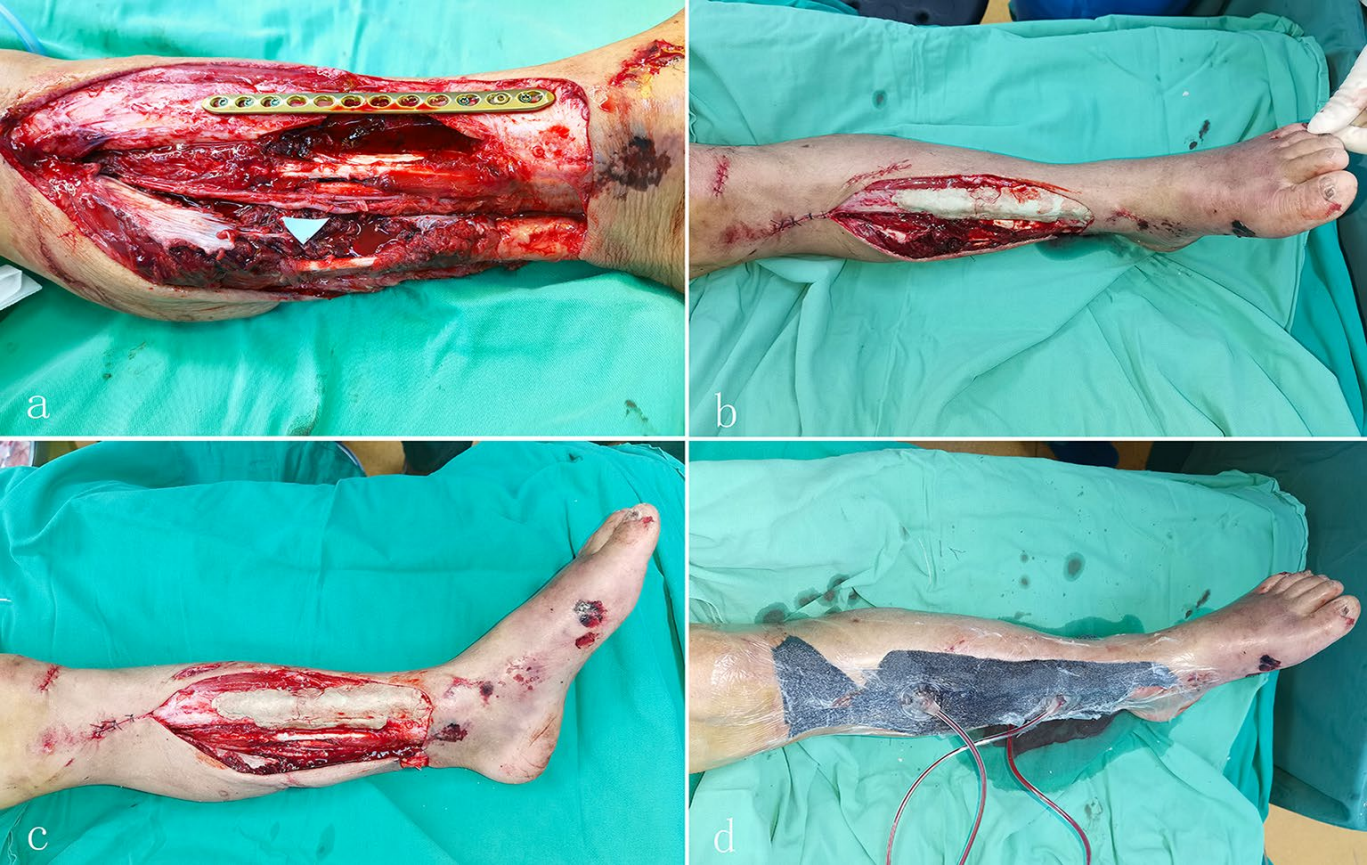
**(a**) Fixation of the bone fracture with a locking plate. (**b**) and **(c**) The bone defect and surface of the locking plate were completely covered with antibiotic PMMA cement. (**d**) The remaining wounds were covered using a vacuum-sealing drainage.

The treatment process was divided into three steps. In the first stage, at the emergency department, intravenous second-generation cephalosporin antibiotics^9,10^ (cefuroxime) were administered as soon as possible after the patient entered the hospital. Initial debridement of the devitalised and contaminated tissues was performed by a senior doctor using loupe magnification, tourniquet control, and irrigation with 9 L of solution. Temporary internal fixation was performed with a 3.5-mm system antibiotic cement-coated locking plate. Internal fixation was performed with a locking compression plate, locked with 2–3 screws at each end (Synthes, Solothurn, Switzerland). The plate and screws were completely covered with antibiotic polymethyl methacrylate (PMMA) cement (PALACOS®, Heraeus, Hanau, Germany). The cement was premixed with 5 g vancomycin added to 40 g gentamicin cement containing 0.5 g gentamicin. The coverage with antibiotic PMMA needs to be at least 1 cm beyond the locking plate and about 0.5 cm thick. Of these patients, 10 free tibial fragments were removed, resulting in segmental bone defects ranging between 3 and 9 cm in length. The bone defects were filled with antibiotic PMMA cement. All fibular fractures were fixed internally during this stage, and the remaining wounds were covered using a vacuum-sealing drainage (Wuhan VSD Medical Science & Technology Co., Ltd., Wuhan, China) (Fig. 1a–d).

After 1–7 (mean, 4.3) days, during the second stage, all patients underwent terminal internal fixation and replacement of the antibiotic-loaded bone cement. Internal fixation was performed using anatomically-locked compression plates, and the original locking plate was maintained; however, the surface antibiotic bone cement was replaced with new antibiotic PMMA cement. Ten cases of free bone fragment removal, treated using the Masquelet technique, were further filled with antibiotic-loaded bone cement. In this stage, free anterolateral thigh (ALT) flap reconstruction was also performed. The ALT flaps ranged from 10 to 33 cm in length and 5 to 16 cm in width.

During the third stage (6–32 weeks after trauma; mean, 11.9 weeks), bone graft surgery was performed in patients with bone defects treated using the Masquelet technique. White blood cell count, erythrocyte sedimentation rate, and C-reactive protein levels were tested every 2 weeks before surgery for two months. Thereafter, the bone cement was removed, and the defect was filled with autogenic cancellous bone.

## Results

All transferred ALT flaps successfully covered the wound and survived without any incidents. Two (10%) patients developed a wound infection after the second stage, of whom one was re-admitted to the operating room for debridement. One (5%) patient developed severe bone infection 3 months after three-stage bone graft surgery. Debridement was performed again, and all internal fixators and transplanted cancellous bone were removed. After partial thorough debridement, Ilizarov osteotomy was performed for transport. The bone healed after 8 months. In 15 cases, the flap donor site was closed directly, while skin grafting was required in 5 cases; the skin grafts survived without incident, and donor sites healed without scar hyperplasia or scar pain. The length of hospital stay ranged from 8 to 50 (mean, 21.5) days. The mean follow-up period after bone grafting was 17.8 (range, 12–54) months, and bone union was achieved within 4–7 months.

### Case example

**Figure 2:**
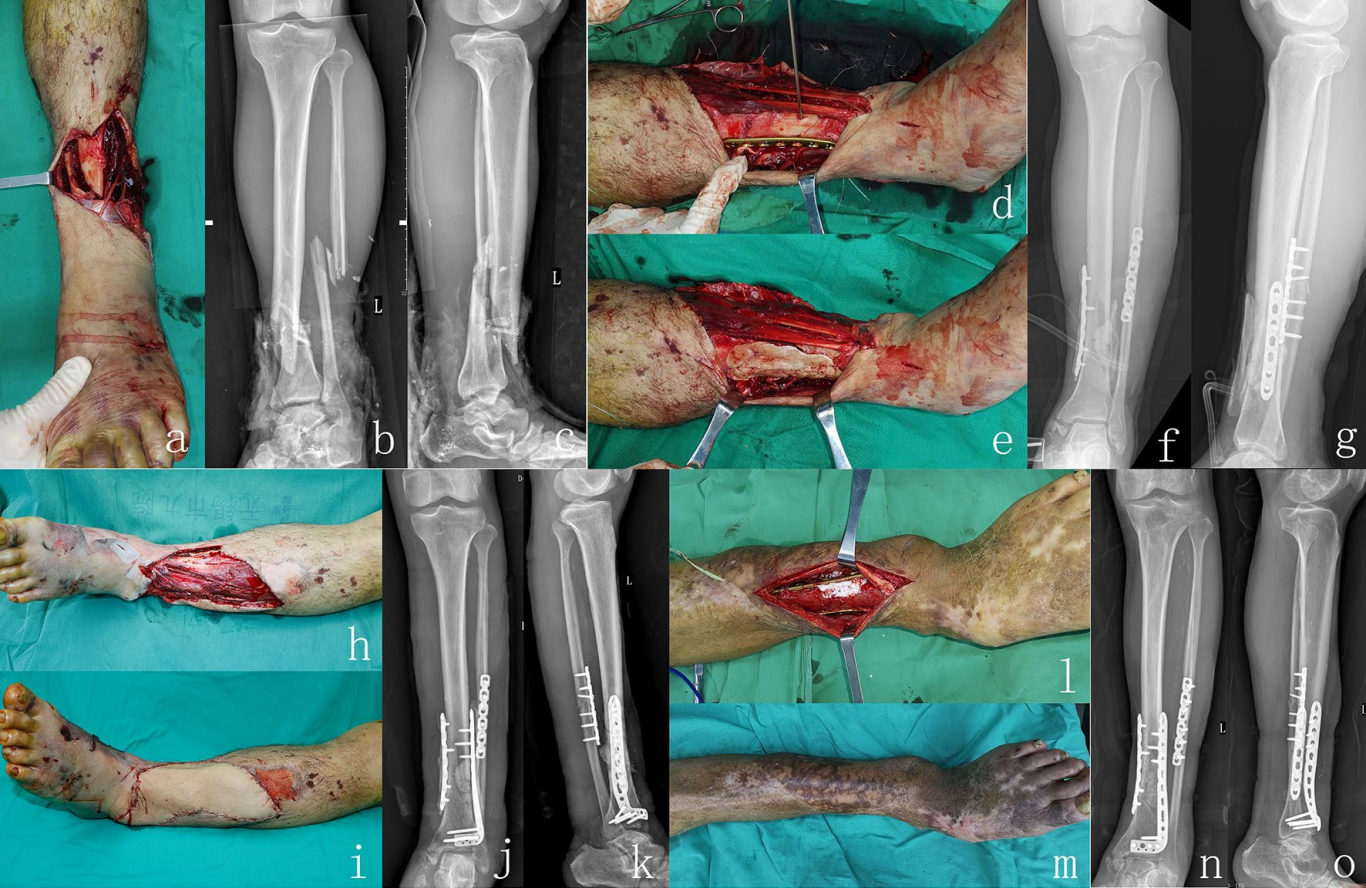
An example of an injury in a 64-year-old male patient. (**a**) Visual inspection of the injury showing anterior and lateral wounds on the lower part of the left. (**b**) Anteroposterior (AP) radiographs showing comminuted fractures in the middle and lower part of the tibia and the middle part of the fibula. (**c**) lateral radiographs of the fracture. (**d**) Fixation of the fracture with the locking plate. (**e**) The surface of the locking plate was completely covered with antibiotic PMMA cement. (**f**) and (**g**) The patient’s AP and lateral radiographs showing the fracture is fixed by antibiotic cement-coated locking plate. (**h**) The irregular defect was located on the patient’s left side. (**i**) A free flap covering the reconstructed soft-tissue. (**j**) and (**k**) The patient’s AP and lateral radiographs showing fixation of the fracture with an antibiotic cement-coated locking plate and anatomically-locked compression plate. (**l**) the bone cement was removed, and the defect was filled with autogenic cancellous bone. (**m**) appearance at 14-month follow-up. (**n**) and **(o**): The AP and lateral radiographs showed fracture healing at 14 months after surgery. AP, anterior posterior.

A 62-year-old man sustained a traffic accident injury comprising a Gustilo grade IIIB right distal tibial meta-epiphyseal fracture (AO 43C3). During the first stage, debridement was performed, and the wound was irrigated with 9 L of solution. All free bone fragments were removed, the tibia length was maintained, and an antibiotic cement-coated locking plate was attached. The second stage was initiated 2 days after noting improvements of the patient’s general condition, such as good mental state and no obvious anaemia (haemoglobin ≥ 100 g/L) or hypoproteinaemia (albumin 35 g/L). Preoperative computed tomography angiography and color Doppler sonography were applied to locate the perforator of the ALT flap, which was transferred without the fascia lata, to repair the wound. The tibial defect was filled with antibiotic-loaded bone cement and stabilised with a locking plate. The flap measured 26 × 8 cm, and the bone defect was 5 cm in length. The third stage was performed 13 weeks after trauma to remove the bone cement and fill the defect with autogenic cancellous bone. Six months following trauma, the graft appeared to be completely integrated. The patient was satisfied with the outcome and reported no pain during walking over the following 22 months (Fig. 2).

## Discussion

Open fractures of the tibia and fibula account for up to 40% of all open fractures^11^. Due to the special anatomical location, severe high-energy injuries may cause extensive bone and soft tissue defects. Gustilo IIIB and IIIC wounds are seriously polluted, accompanied by bone and soft tissue defects, vascular and nerve damage, postoperative wound and bone infection, and limb necrosis. The risk of amputation is high, and the infection rate can reach as high as 63%^12^. Dealing with such injuries remains difficult for clinicians. Emergency treatment of open fractures is particularly important, as it lays the foundation for subsequent treatment, can even determine whether the patient's limb can be salvaged in the later stage, and can further influence the degree of functional recovery after limb salvage. Emergency treatment primarily involves bone fixation and soft tissue treatment. Temporary fixation with an external fixator remains the first choice for emergency fixation of open tibial fractures^13,14^. Limitations of external fixation, such as loosening of the bracket, infection of the needle tract, poor stability, angulation of fractures, and malunion, can all affect muscle contraction and joints with a large range of motion^15,16^. Further, although an external fixation frame can be used as a temporary fixation option, it is not the best choice as it often causes difficulties in soft tissue repair and delays wound closure.

Although internal fixation avoids the disadvantages of external fixation, it is prone to deep infections. To reduce the infection caused by implants, researchers worldwide have explored the use of antibiotic cement covering to prevent the direct adhesion of bacteria and avoid the formation of bacterial biofilms, thereby controlling the incidence of infection. Studies in the literature have further shown that applying antibiotic cement to cover the surface of the implant can effectively prevent bacteria from adhering to the surface of the implant following direct contact. At the same time, the antibiotic bone cement can kill bacteria adhering to the surface and surrounding tissues by releasing a high local concentration of antibiotics, to provide a “self-cleaning effect” for internal fixation^17^. Antibiotic bone-cement-coated internal fixation has been successfully applied in the clinical treatment of bone infections. Wang retrospectively analysed 548 cases of antibiotic bone cement plate treatment for infected bone defects, finding an infection control rate of 97.6%^18^. In the present study, the authors proposed the concept of ‘Chongqing technology’, that is, the use of antibiotic bone cement plate composite structure internal fixation to treat bone infection. It is believed that thorough debridement can allow the conversion of infected wounds into only polluted wounds. Internal fixation with antibiotic bone cement can subsequently be used in polluted wounds; in such wounds, antibiotics are released through local high concentrations of antibiotic bone cement, and the postoperative standardised use of antibiotics can be applied to convert polluted wounds into clean wounds. Therefore, in our study, antibiotic-loaded cement-coated plates were used as emergency fixation methods for open tibial and fibular fractures. In the emergency department, a contaminated wound surface can be transformed into a clean one through thorough debridement. In the present study, the bacterial environment and the free flap technique were applied to completely repair the wound within 7 days, and terminal internal fixation was added or replaced at the same time. The infection rate was significantly reduced, and the deep infection rate was 5%.

In previous studies in the literature, the infection rate of emergency external fixation, secondary flap coverage, and replacement of internal fixation ranged 14–27%^15,19–21^, while the infection rate of emergency flap transplantation combined with internal fixation treatment was 9.2%^22^. This indicates that the use of antibiotic-loaded bone cement-coated steel plate internal fixation does not lead to a higher infection rate, has a lower infection rate than conventional surgery, and avoids wearing discomfort caused by the emergency use of external fixators, needle tract infection, and other disadvantages. The average length of hospitalisation of patients in this study was 19.9 days, which was significantly shorter than the average of 39 days reported in the literature^23^.

Herein, we demonstrate the viability of using an antibiotic-loaded cement-coated locking plate in the emergency department for treating open tibial and fibular fractures. There are limitations to the use of antibiotic-loaded cement-coated locking plates in patients. The following points should be considered when applying an antibiotic-loaded bone-cement locking plate in the emergency department for open fractures. First, the emergency wound must be debrided thoroughly, and the conversion of a “contaminated wound” into a “clean wound” must be performed as the basis for the application of an antibiotic bone cement plate. Second, follow-up treatment strategies should be formulated during the initial assessment to ensure that the wound can be completely repaired within 7 days, and the occurrence of infection can be effectively controlled. Third, it is necessary to standardise the application of intravenous antibiotics for 3 days following internal fixation and prevent subsequent infection using local high-concentration antibiotics and systemic intravenous antibiotics.

Based on our experience, we reported the initial efficacy of a proposed new treatment model using antibiotic-loaded cement-coated locking plates as primary temporary internal fixation, definitive internal fixation, and free flap grafting within 7 days for Gustilo grade IIIB/C open tibial fractures. This method may offer a better chance of infection eradication and improved recovery of limb function, without increasing the infection recurrence rate. However, this study has several limitations. First, the retrospective nature and small sample size limit the generalisability of the results. Second, the follow-up period was relatively short. In addition, this treatment only considered infection control, and some cases required reconstruction of the bone defects at a later stage; thus, the long-term treatment outcome of this method needs further investigation and a prospective randomized controlled study. We look forward to providing further novel ideas and technologies for emergency fixation of open tibial fractures.

## Data Availability

The data generated and analysed during this study are available from the corresponding author on reasonable request.

## References

[1] Bowen TR, Widmaier JC (2005). Host classification predicts infection after open fracture. Clin. Orthop. Relat. Res..

[2] Minehara H (2023). Open fractures: Current treatment perspective. OTA Int..

[3] Diwan A, Eberlin KR, Smith RM (2018). The principles and practice of open fracture care, 2018. Chin. J. Traumatol..

[4] Kang Y (2020). "Primary free-flap tibial open fracture reconstruction with the Masquelet technique" and internal fixation. Injury.

[5] Dheenadhayalan J, Nagashree V, Devendra A, Velmurugesan PS, Rajasekaran S (2023). Management of open fractures: A narrative review. J. Clin. Orthop. Trauma.

[6] Clifford RP, Beauchamp CG, Kellam JF, Webb JK, Tile M (1988). Plate fixation of open fractures of the tibia. J. Bone Jt. Surg. Br..

[7] Cho JW (2020). Bone⁃graft resorption reduced by the induced membrane technique and factors affecting volumetric changes: An analysis of 120 serial computed tomographic scans in 40 patients. J. Bone Jt. Surg. Am..

[8] Shen J (2021). Management of surgical site infection post-open reduction and internal fixation for tibial plateau fractures. Bone Jt. Res..

[9] Suzuki T (2023). Type III Gustilo-Anderson open fracture does not justify routine prophylactic Gram-negative antibiotic coverage. Sci. Rep..

[10] Zalavras CG (2017). Prevention of infection in open fractures. Infect. Dis. Clin. North Am..

[11] Wood AM, Robertson GAJ, MacLeod K, Porter A, Court-Brown CM (2017). Epidemiology of open fractures in sport: One centre's 15-year retrospective study. World J. Orthop..

[12] Schenker ML, Yannascoli S, Baldwin KD, Ahn J, Mehta S (2012). Does timing to operative debridement affect infectious complications in open long-bone fractures? A systematic review. J. Bone Jt. Surg. Am..

[13] Zhu YL (2022). Ilizarov technology in China: A historic review of thirty-one years. Int. Orthop..

[14] Khaled A, El-Gebaly O, El-Rosasy M (2022). Masquelet-Ilizarov technique for the management of bone loss post debridement of infected tibial nonunion. Int. Orthop..

[15] Hatashita S (2021). 'Acute Masquelet technique' for reconstructing bone defects of an open lower limb fracture. Eur. J. Trauma Emerg. Surg..

[16] Deng L (2020). The Masquelet technique combined with the muscle flap for use in emergency management of acute Gustilo type III trauma of the lower limb with segmental bone loss: Case series. Int. J. Surg..

[17] Jia C (2020). An antibiotic cement-coated locking plate as a temporary fixation for treatment of infected bone defects: A new method of stabilization. J. Orthop. Surg. Res..

[18] Wang X (2021). Antibiotic cement plate composite structure internal fixation after debridement of bone infection. Sci. Rep..

[19] Costa ML (2018). Effect of negative pressure wound therapy vs standard wound management on 12-month disability among adults with severe open fracture of the lower limb: The WOLLF randomized clinical trial. JAMA.

[20] Messner J (2020). Lower limb paediatric trauma with bone and soft tissue loss: Ortho-plastic management and outcome in a major trauma centre. Injury.

[21] Olesen UK (2015). A review of forty five open tibial fractures covered with free flaps. Analysis of complications, microbiology and prognostic factors. Int. Orthop..

[22] Rajasekaran S (2009). Immediate primary skin closure in type-III A and B open fractures: RESULTS after a MINIMUM of five years. J. Bone Jt. Surg. Br..

[23] Kang Y (2020). Subacute reconstruction using flap transfer for complex defects of the upper extremity. J. Orthop. Surg. Res..

